# Partnerships for Policy Development: A Case Study From Uganda’s Costed Implementation Plan for Family Planning

**DOI:** 10.9745/GHSP-D-15-00300

**Published:** 2016-06-20

**Authors:** Alyson B Lipsky, James N Gribble, Linda Cahaelen, Suneeta Sharma

**Affiliations:** a RTI International, Health Policy Project, Washington, DC, USA; b Palladium, Health Policy Project, Washington, DC, USA; c U.S. Agency for International Development, Washington, DC, USA

## Abstract

In global health, partnerships between practitioners and policy makers facilitate stakeholders in jointly addressing those issues that require multiple perspectives for developing, implementing, and evaluating plans, strategies, and programs. For family planning, costed implementation plans (CIPs) are developed through a strategic government-led consultative process that results in a detailed plan for program activities and an estimate of the funding required to achieve an established set of goals. Since 2009, many countries have developed CIPs. Conventionally, the CIP approach has not been defined with partnerships as a focal point; nevertheless, cooperation between key stakeholders is vital to CIP development and execution. Uganda launched a CIP in November 2014, thus providing an opportunity to examine the process through a partnership lens. This article describes Uganda’s CIP development process in detail, grounded in a framework for assessing partnerships, and provides the findings from 22 key informant interviews. Findings reveal strengths in Uganda’s CIP development process, such as willingness to adapt and strong senior management support. However, the evaluation also highlighted challenges, including district health officers (DHOs), who are a key group of implementers, feeling excluded from the development process. There was also a lack of planning around long-term partnership practices that could help address anticipated execution challenges. The authors recommend that future CIP development efforts use a long-term partnership strategy that fosters accountability by encompassing both the short-term goal of developing the CIP and the longer-term goal of achieving the CIP objectives. Although this study focused on Uganda’s CIP for family planning, its lessons have implications for any policy or strategy development efforts that require multiple stakeholders to ensure successful execution.

## Abstract (precis)

The development and launch of the costed implementation plan (CIP) in Uganda was successful in many ways. However, it would have benefitted from more focus on long-term partnership development critical for executing the CIP and by including district health officers—key players in executing the plan—more substantially in the process. Using a partnership approach sets the stage for ensuring that the right people are contributing to both development and execution.

## INTRODUCTION

Partnerships between practitioners and policy makers are a necessary strategy to address complex challenges that require multiple stakeholders to work together toward the same goal,^1^^,^^2^ including the development and execution of global health policies and programs such as national costed implementation plans (CIPs).^1^^,^^3^ CIPs are planning and management tools, developed through a government-led consultative approach, that detail the activities needed over multiple years, executed by a range of organizations, to meet program goals as well as the costs associated with the activities.^4^

Since 2009, many countries have used CIPs as an approach to create a multiyear map designed to help governments achieve their family planning goals. Specifically, a CIP can help determine the human, financial, material, and technical resources needed, and it can be used to justify resource mobilization.^5^ Ideally, CIPs effect change by translating broad family planning goals into “concrete programs and policies.”^5^ CIPs aim to help countries unify stakeholders behind this strategy, raise the profile of family planning, and leverage financial and technical resources from multiple stakeholders. Family planning leaders in Uganda launched their first CIP in November 2014 after conducting a vibrant consultative process.^6^ This process provided a unique opportunity to examine the development of a CIP through the lens of partnership.

Costed implementation plans (CIPs) translate broad family planning goals into concrete programs and policies.

This article applies a partnership evaluation framework to assess the extent to which Uganda’s CIP development process depended on stakeholder engagement and commitment to shared goals. It pushes the CIP development process, which was born out of strategic planning and budgeting processes,^7^ to another level, seeking to understand whether a partnership approach might strengthen both the development process and the subsequent execution. It thus looks at the presence of partnership factors and their effect on partners’ perceptions of their work. The article also explores how these factors might affect execution and provides guidance to those embarking on CIP development in other countries.

This study aimed to understand whether a partnership approach could strengthen both the development process of CIPs and their subsequent execution.

We begin the article by introducing a definition of partnership from the literature, then briefly explain what CIPs are. We used a partnership evaluation framework as the methodology to analyze the CIP process, examine the Ugandan context and CIP development, discuss the findings from the research, and make recommendations for how CIP development and execution can be strengthened when treated as a partnership. We conclude by discussing the implications of using a partnership framework more explicitly as practitioners develop family planning CIPs and other types of health strategies and policies.

## DEFINING PARTNERSHIPS AND COSTED IMPLEMENTATION PLANS

Partnerships take many forms, making it difficult to develop one single definition. While there is a variety of partnership definitions in the literature (for example, see Buse and Walt, 2000^8^), generally partnerships can be defined as “joint initiatives between the public sector, nongovernmental organizations and the corporate sector.”^8^ Partnerships can be further defined according to 2 dimensions: mutuality and organization identity.^9^ Mutuality refers to how partners rely on each other to advance a common cause. Mutuality does not presume that power dynamics are equal between partners, but that each actor has rights and responsibilities that “seek to maximize benefits for each party.”^9^ Organization identity, on the other hand, refers to each organization’s unique traits in the partnership, especially the organizations’ ability to maintain their core mission and values over the long term.^9^

Often partnership definitions include such factors as having a degree of reciprocity between partners, clear objectives, and mutual responsibilities to advance shared interests.^7^ These shared interests are limited only by one’s imagination and can include service delivery, infrastructure development, capacity building, economic development, and policy.^10^ Policy partnerships between governments and NGOs are those that “design, advocate for, coordinate, or monitor public policies. Policy partnership structures can vary from informal issue-specific networks to formal cross-sectoral committees, task forces, or special commissions.”^10^

Partnerships can be critical for policy or strategy execution. Within family planning, in 2004 the U.S. Agency for International Development (USAID) launched and promoted the “Strategic Pathway to Reproductive Health Commodity Security” (SPARHCS) to improve availability of and access to commodities. The approach includes partnership guidelines for working across government, the private sector, and donors not only to develop a strategy but also to execute the strategy.^11^ SPARCHS was successfully used in many countries to plan, prioritize, and execute strategies to improve access to reproductive health commodities.^11^ A number of global family planning partnerships have been formed in recent years, such as the global Family Planning 2020 (FP2020) partnership and the Ouagadougou Partnership. However, there has been little exploration of family planning policy partnerships at the country level. The CIP development process is an example of one such partnership.

CIPs for family planning are a recent development that evolved out of a perceived need to unify diverse stakeholders around a shared strategy to achieve family planning goals at a national or subnational level. Since 2009, at least 20 countries have developed CIPs.^12^ In most cases, they have followed a common systematic approach, including establishing a national task force and following a 10-step, customizable process (Box 1).^4^^,^^13^ FP2020 led a global effort to develop a standardized approach to crafting CIPs and engaged those international organizations that have been developing CIPs since 2009.

CIPs evolved out of a need to unify diverse stakeholders around a shared strategy.

**BOX 1. Typical 10-Step Costed Implementation Plan (CIP) Development Process**

**PHASE I: PLAN**

Step 1: Obtain government and key stakeholder buy-in.
Step 2: Detail roadmap and secure resources for CIP development.

**PHASE II: DEVELOP**

Step 3: Conduct a family planning situational analysis.
Step 4: Detail and describe a technical strategy with sub-activities and timeline.
Step 5: Estimate resources and costs.
Step 6: Identify financing gaps.
Step 7: Secure final approval and launch the plan.

**PHASE III: EXECUTE**

Step 8: Set up and manage institutional arrangements for implementation.
Step 9: Design and implement performance-monitoring mechanisms.
Step 10: Develop and implement a resource mobilization plan.

Source: Health Policy Project 2015.^7^

Although the CIP development process is not explicitly promoted as a partnership, the development process requires mutuality and organization identity, and thus is in practice a partnership. The outcomes of partnerships depend on more than just funding and technical inputs; they are affected by “the institutions and incentives governing the execution of policies and programs, including informal rules, regulations, controls, and structures.”^9^ Thus, using a partnership approach to analyze CIP development can provide useful insights to help ensure that the partnership itself is well positioned to apply the CIP and contribute to the achievement of FP2020 goals.

## FRAMEWORK FOR EVALUATING PARTNERSHIPS

The purpose of this evaluation was to look at the rules of engagement and relationships within the partnership that developed Uganda’s CIP. Capturing the complexity of partnership requires a multifaceted evaluation methodology. Our analysis draws on part of a framework developed to assess a global family planning consortium (personal communication with Dr. Jennifer Brinkerhoff, Professor of Public Administration and International Affairs, The George Washington University, January 2015) and identifies several categories of factors that affect partnership effectiveness. The full framework outlines 5 overarching categories on which to assess partnerships^9^:

1. Presence of prerequisites and success factors
2. Degree of partnership
3. Outcomes of the partnership relationship
4. Partner performance
5. Efficiency and strategy

Because this study looked only at the CIP development process, we focused on the first category of factors—presence of prerequisites and success factors (Box 2). Nonetheless, we included one additional factor—ownership—later as an area of analysis after it emerged as a theme in the key informant interviews. Although ownership is not included in Brinkerhoff’s partnership evaluation framework, it has been cited^14^ as important for policy development, execution, and sustainability; thus, we added it to our framework.

**BOX 2. Presence of Prerequisites and Success Factors Affecting Partnership Effectiveness**

1. Perceptions of partners’ tolerance for sharing power^9^
2. Partners’ willingness to adapt to meet the partnership’s needs: perception of receptivity to new solutions to improve the partnership, its value, and day-to-day performance; speed and flexibility in addressing the need for corrective action; accommodation of special requests among the partners; responsiveness of partners to unforeseen situations^9^
3. Existence of partnership champions: existence of champions within each partner organization and within the partnership as a whole; focus of champions’ advocacy (internal to a partner organization, within the partnership, externally)^9^
4. Trust: character-based perceptions of integrity, honesty, moral character, reliability, confidentiality, as appropriate, etc.; competence-based perceptions of competence in prescribed/assumed skill areas, understanding of partnership, etc.^9^
5. Confidence: use of standard operating procedures, contractual agreements, and their degree of formality^9^
6. Senior management support: direct participation; provision of resources and support to organization members participating in the partnership^9^
7. Ability to meet performance expectations: function of both external constraints and partner capacity^9^
8. Clear goals: consistent identification of partnership goals and mission; regular partner meetings to review, revise, and assess progress in meeting identified goals; shared common vision for the partnership; mutually determined and agreed-upon partnership goals^9^
9. Partner compatibility: knowledge and understanding of partners’ missions, operations, and constraints; previous conflict or confrontations among partners; compatible operating cultures (e.g., operating philosophies, management styles, teamwork); compatible constituencies; compatible core values; mechanisms to address incompatibilities^9^
10. Conflict: degree; frequency; extent of conflict avoidance within partnership; presence/absence of one or more dominating partners^9^
11. Ownership: degree to which in-country stakeholders are perceived to be responsible and able to manage the development and implementation of a policy, strategy, or program (emerged as a theme in this study)^14^

## CASE STUDY: UGANDA

Uganda developed its CIP over several months in 2014, and the final CIP was launched at a national event in November 2014,^6^ with a start date of July 2015.^15^ Published by the Ministry of Health (MOH), the CIP identifies 5 priorities (Box 3), and it is estimated to cost US$236 million between 2015 and 2020. If executed as intended, the activities in the CIP will increase the number of women in Uganda using modern contraception from approximately 1.7 million in 2014 to 3.7 million in 2020.^16^

**BOX 3. Uganda Costed Implementation Plan (CIP) Priorities**

1. Priority #1: Increase age-appropriate information about, access to, and use of family planning among young people ages 10–24 years.
2. Priority #2: Promote and nurture change in social and individual behavior to address myths, misconceptions, and side effects, and improve acceptance and continued use of family planning to prevent unintended pregnancies.
3. Priority #3: Implement task sharing to increase access, especially for rural and underserved populations.
4. Priority #4: Carry out mainstream implementation of family planning policy, interventions, and delivery of services in multisectoral domains to facilitate a holistic contribution to social and economic transformation.
5. Priority #5: Improve forecasting, procurement, and distribution of commodities, and ensure full financing for commodity security in the public and private sectors.

Source: Uganda Ministry of Health, 2014.^15^

Uganda was selected as a case study for examining CIPs in a partnership framework for several reasons. First, Uganda has a strong enabling environment for family planning and CIPs. Second, it followed the commonly used 10-step CIP process, including establishing a CIP task force. Additionally, its CIP development process included a particularly robust consultative process. Moreover, because the timing was right, Uganda provided an opportunity to assess partnership prerequisites and success factors present during the CIP development process.

### Strong Enabling Environment

Uganda’s 2011 Demographic and Health Survey (DHS) highlighted the country’s high unmet need for family planning.^17^ As early as 2012, President Museveni and other high-profile officials at the national level newly expressed strong public support for family planning.^15^ The FP2020 global initiative that resulted from the 2012 London Summit on Family Planning provided a structure within which Uganda could set ambitious goals. At the London Summit, Uganda committed to reducing unmet need for family planning from 40% to 10% by 2022 by improving program and service delivery, increasing financial commitments and expenditures, and addressing policy and political challenges.^15^ As a result of these commitments, the Ugandan government was obligated to take action on family planning. Finally, local pressure to develop a comprehensive national plan for family planning was growing—discussions between the government, civil society, and international partners started as early as May 2013 and culminated in a national meeting in September 2013 at which a leading coalition of family planning service providers called for the MOH to develop a national family planning strategy. Within the region, Kenya, Tanzania, and Zambia had already adopted CIPs,^18^^-^^20^ and donors were prepared to provide support in developing a CIP in Uganda. Then, in early 2014, one donor provided initial funding for the development of a national plan for family planning before the CIP development process officially began.^15^

### Development of a Task Force as Part of the CIP 10-Step Process

Although not all countries establish task forces to develop CIPs, Uganda did take this approach. As part of Step 1 of the 10-step process (Box 1), Uganda’s MOH established and chaired a CIP task force in May 2014 comprising major family planning stakeholders—donors, advocates, and implementing partners. Specifically, the task force included the MOH and national-level stakeholders, such as the Partners in Population and Development–Africa Regional Office; Uganda Family Planning Consortium; Reproductive Health Uganda; Program for Accessible Health, Communication and Education; Uganda Health Marketing Group; the United Nations Population Fund (UNFPA); and USAID. Establishing the CIP task force reflected the importance of organization identity; a strong CIP requires different perspectives. Further, some level of mutuality was needed because of the important role NGOs and donors play in family planning service delivery in Uganda. Thus, the Ugandan government could not be the only organization selecting activities for inclusion in the CIP.

Uganda’s MOH established and chaired a CIP task force comprising major family planning stakeholders.

In June 2014, the MOH officially requested donor support from both multilateral and bilateral funding agencies for the CIP development process. Donor support was committed in June 2014, and the task force formed the technical support team (TST), which included a Ugandan consultant and support from 2 international partners. The TST developed a roadmap, and shared it with the task force for approval. Throughout CIP development, the task force was responsible for managing the process, including holding meetings and workshops, making overall strategy decisions, approving deliverables, facilitating approval of the CIP itself, and coordinating actors at the national and subnational levels. The TST was responsible for technical work, including drafting documents for the task force to review and approve.

### Engaging in a Vibrant Consultative Process

As part of engaging a range of stakeholders, the TST held a series of more than 30 national and subnational consultations between July and August 2014, focusing on 9 technical areas. To determine the technical areas for the consultations, the TST proposed several areas to the task force based on global best practices, Uganda’s key priorities, and data on the current family planning and policy landscape in the country. The task force made the final decision on which areas the consultations would address. The selected technical areas comprised^15^:

- Contraceptive security
- Human resources
- Health systems management
- Advocacy
- Social and behavior change communication
- General family planning service delivery
- Youth-friendly family planning/reproductive health services
- Integration of family planning services into other health services and sectors
- Decentralization

Each consultation followed a similar structure that fostered active participation: presentations on the technical area, followed by group work to identify family planning priorities and practical solutions to achieving them. Participants elected representatives to serve as points of contact for the task force and the TST as work on the CIP moved forward. To solicit local views of family planning challenges, the TST also held subnational consultations. In addition, the TST held focus group discussions with youth, DHOs, development partners, Government of Uganda sector ministries, and local community members, but these stakeholders were not part of the technical consultations.

The CIP was developed through an iterative process between the TST, the task force, and the representatives elected as part of the consultation process. As data from the consultations came in, the TST created a draft activity list that the technical area experts reviewed using a simple prioritization tool to assess each activity’s potential impact and feasibility and to finalize the CIP’s priorities (Box 3). Once the CIP priorities and the activity list were finalized, the TST completed the costing using a Microsoft Excel-based tool (available at www.familyplanning2020.org), and the CIP was drafted. The task force reviewed the activity list and CIP throughout the drafting process.

Uganda’s CIP was developed through an iterative and collaborative process.

## EVALUATION METHODS

After selecting the partnership evaluation framework, we reviewed the TST’s published and unpublished project documents; government reports, such as the most recent DHS and the CIP; and other materials regarding family planning services in Uganda. Using a deductive qualitative approach to assess the Uganda CIP process, we developed a semi-structured interview guide to assess the 10 partnership prerequisites and success factors outlined in the evaluation framework. We used the interview guide with both Ugandan and international stakeholders. The interview guide asked key informants about their experiences and perceptions regarding partnership and the CIP process, including probes for follow-up. The Futures Group’s Internal Research Review Committee reviewed and approved the study’s research protocol.

One of the authors and a consultant on the evaluation conducted 22 in-depth interviews, with informants sampled in a 2-stage process. The first stage took place in February and March 2015, and was a purposeful sample consisting of 12 task force and TST members. The researchers then asked these informants to identify individuals who were not task force members but were peripherally involved in developing the CIP and important for CIP execution. Based on recommendations from the informants, the interviewers spoke with representatives from 3 faith-based organizations (FBOs) and 7 DHOs (1 representative from each organization/office) in June 2015. The interviewers did not conduct interviews with youth, local community members, or Government of Uganda sector ministries. The sampling method produced a minimum sample based on expected reasonable coverage.

The interviews were conducted via phone, Skype, or in person; they lasted between 30 minutes and 1 hour. The interviewers took notes, and we supplemented the interviews with audio recordings when possible. We did not transcribe the audio recordings. All the key informants gave verbal permission to participate in the assessment and to be recorded when applicable. All interviews were confidential, with identifying information removed from the interview notes and summaries.

We developed the primary codes for analysis according to the selected evaluation framework. We then read and coded the notes. After the initial coding, we read the notes again to determine what, if any, additional codes were needed. Once coding was completed, we reviewed data and identified themes.

This evaluation and the framework itself are focused on the inner workings of the partnership, not necessarily on contextual issues—in this case, broader issues within Uganda that might affect the partnership’s ability to achieve its goals (e.g., participation, inclusion, transparency, accountability). The framework includes external constraints as a factor; however, this evaluation was limited to key informant interviews, meaning that if the evaluation participants did not mention a constraint, it is not represented in the findings.

## FINDINGS

The findings reveal that participants’ perspectives were often aligned with how involved they were in the CIP development process and their relationship to the task force. The CIP development process involved 3 key types of relationships: (1) relationships within the task force itself, (2) relationships between the task force and the TST, and (3) relationships between the task force and the people who participated in the consultations. The Table summarizes the degree to which each prerequisite and success factor was present in the 3 main relationship types in the CIP development process. Several partnership factors had high presence, one factor had low presence (which was positive since the factor pertained to conflict), and several had mixed representation across the 3 relationships.

**TABLE:** Evaluation of Partnership Success Factors and Prerequisites for the 2014 Uganda Costed Implementation Plan by Types of Relationships

	Types of Relationships		
Factors	Within the CIP Task Force	Between CIP Task Force and TST	Between CIP Task Force and Consultation Participants
**Factors with high presence across all 3 relationships**			
Partners’ willingness to adapt to meet partnership’s needs	High	High	High
Existence of partnership champions	High	High	High
Ability to meet performance expectations	High	High	High
Clear goals	High	High	High
Senior management support	High	N/A	High
Partner compatibility	High	High	High
**Factors with low presence across all 3 relationships**			
Conflict (degree, frequency, conflict avoidance, dominating partner)	Low	Low	Low
**Factors with mixed presence across the 3 relationships**			
Perception of partners’ tolerance for sharing power	Low	Medium	Low
Trust	High	Medium	Medium
Confidence in procedures	Low	Medium	Low
Ownership	High	High	Low

Abbreviations: CIP, costed implementation plan; NA, not applicable; TST, technical support team.

### Partnership Factors With High Presence Across All 3 Relationships

Factors present to a high degree in all 3 relationships consisted of: (1) partners’ willingness to adapt to meet the partnership’s needs, (2) existence of partnership champions, (3) ability to meet performance expectations, (4) clear goals, (5) senior management support, and (6) partner compatibility.

**Partners’ willingness to adapt to meet the partnership’s needs.** Adaptability across all 3 relationships was characterized by task force members’ and consultation participants’ willingness to learn from one another about priorities and differences in technical approaches. During task force meetings and the consultations, the majority of the informants noted a culture of learning that allowed partners to have differences of opinions without halting the process. Lively discussions at task force meetings, between the task force and the TST, and during the national and subnational consultations exemplified this culture of learning, allowing for different perspectives to be aired and deliberated (Box 4).

**BOX 4. What Did Willingness to Adapt Look Like When Developing the 2014 Uganda Costed Implementation Plan?**

1. Task force and technical support team (TST) members disagreed about the necessity of subnational consultations, yet after discussing their respective concerns, the subnational consultations were held.
2. Two international partners on the TST previously had used slightly different methodologies for supporting costed implementation plan (CIP) development. TST members reconciled their approaches and took principles from each to ensure the CIP was developed efficiently while meeting Uganda’s needs.
3. During the national and subnational consultations, information flowed freely. Faith-based organizations, in particular, appreciated the opportunity to dispel some myths around natural family planning.
4. During the national and subnational consultations, implementers were given an opportunity to discuss policy barriers hindering certain activities that might be helpful, such as youth-friendly services. In return, policy makers had an opportunity to address the challenges that implementers face and identify ways to address them.

**Existence of partnership champions.** Some high-level champions took an extraordinary interest in supporting and promoting Uganda’s CIP in different ways. For example, advocacy champions were successful in getting the CIP on the agenda in the first place. They came from NGOs and donors—and some champions ended up serving on the task force. After the task force had been established, key informant interviewees stated that there was no consensus that the group should focus on developing a CIP specifically. However, national-level champions serving on the task force convinced the other task force members that a CIP would be the best approach because it would both serve as a national family planning policy and estimate the cost of conducting key activities. Champions among the donors serving on the task force were able to secure the requisite funding for the CIP’s development. Task force members stated there were also champions who ensured the technical quality of the CIP—numerous key informants noted the importance of the TST and one of the donors in this area.

**Ability to meet performance expectations.** Most key informants indicated that they had been able to meet performance expectations regarding CIP *development*. However, all key informants reported having concerns about meeting performance expectations during the CIP’s *execution* phase. They recognized that, first and foremost, they needed to secure new funding and that funding mechanisms needed to be established. They concluded that the MOH will need strong long-term leadership capacity to drive the performance and management of the CIP’s execution. Additionally, to actually execute the activities in the CIP, partners will need to strengthen capacity, especially the capacity to work across sectors, since the CIP calls for a multisectoral approach. Although informants identified some external constraints, such as the brief enactment of Uganda’s Anti-Homosexuality Act in 2014, they expect external constraints to play a larger role during the execution phase. For example, one informant noted that as Uganda gears up for the 2016 elections, it will be increasingly difficult to sustain the attention of politicians and government officials.

Surveyed CIP participants agreed that the MOH needs long-term leadership capacity to drive successful execution of the Uganda CIP.

**Clear goals.** The clear, overarching goal for the task force and the TST was to develop and launch a CIP. Task force and TST key informants noted that the second step in the CIP process—developing a roadmap—helped in clarifying more specific goals for the task force and the TST. For example, the task force was responsible for approving the concept note and terms of reference for the CIP development process, whereas the TST was responsible for data collection, analysis, and drafting. The task force members interviewed agreed that the process needed to emphasize stakeholder engagement to ensure the CIP included their priorities.

**Senior management support.** A high degree of senior management support existed at the national level. Informants from the task force reported that most of the task force participants were the senior management from their respective organizations; these task force members provided strategy, funding, and oversight. The MOH task force participants provided overall guidance and kept MOH leadership aware of CIP progress. Additionally, the MOH assigned people from the reproductive health office to work with the task force and the TST, and 2 staff from the budget and planning department to work with the TST. Informants noted that some senior management from partner organizations played a key role at the inception stage by making sure that the task force was formed and achieving consensus for the CIP. Donor partner task force members were able to secure funds (e.g., funds to hire the national consultant, hold the subnational consultations, and support the launch). Some senior management, in addition to participating in the task force, also met with the TST regularly. Senior management support for consultation participants was largely limited to providing transport funds, yet more participation from senior management was not necessarily expected at this stage.

**Partner compatibility.** At the task force level, partners brought specific skills to the process, and the task force leveraged their comparative advantages. The task force relied on donor partners for their ability to fund the activity, advocacy partners for their technical expertise and history of working for family planning within Uganda, and others for their work with family planning service providers. Further, the perception of compatibility was high because all partners interviewed believed they had the same core values, such as recognizing the importance of family planning. However, even though there was a perception of compatibility during CIP development, many informants anticipate that this compatibility will be tested and perhaps strained once they begin to execute the CIP, especially as NGO implementers begin to compete for funds.

### Partnership Factor With Low Presence Across All 3 Relationships

**Conflict.** Informants reported little conflict during CIP development but anticipate more during CIP execution. Throughout the CIP process, informants noted moments of tension and disagreement but no significant conflict. Task force and TST members stated that there was some tension while the task force was preparing the launch, as some partners who worked in similar areas jockeyed for greater visibility at the launch. Interviewees reported that the MOH stepped in and made the final decisions about visibility. Informants also reported tension when the task force was not prepared to pay per diems or provide transport funds at some consultations. The MOH worked with one of the donors represented on the task force to secure the needed funds. Conflicts over technical differences were resolved amicably by working through the task force, enabled by the culture of learning described earlier.

### Partnership Factors With Mixed Representation Across the 3 Relationships

The partnership factors that had mixed representation across the 3 relationships consisted of: (1) perception of partners’ tolerance for sharing power, (2) trust, (3) confidence, and (4) ownership.

**Perception of partners’ tolerance for sharing power.** Some tolerance for sharing power was present between the task force and the TST. Interviewees from the TST agreed that although much responsibility was delegated to the TST, it reported to the task force and ultimately to the MOH. The data indicate that whereas there was a high level of participation by task force members, the TST, and the consultation participants, power remained with the MOH. Interviewees noted that the MOH was open to solutions proposed by partners, including recommendations that came out of the consultations. However, interviewees also perceived that because Uganda’s CIP was going to be owned by the MOH, the MOH also would exercise ultimate authority, including making final decisions regarding the technical components present in the CIP.

Moreover, during the CIP process, although the majority of funding came from donors, the task force—and the MOH in particular—guided how the resources were used. The task force took advantage of donor funds available for certain activities—in particular, the subnational consultations, which were held because the task force wanted to ensure that the CIP included community consultations. Ultimately a donor was able to make funds available for them.

**Trust.** Trust was strong within the task force and between the task force and the TST. Task force informants indicated a high level of trust within the group due to previous working relationships among task force members and the preparatory work they had done together. Their joint experience meant that members had realistic expectations for each organization’s capacity and the role of each partner. Furthermore, task force members trusted each other’s commitment to the mission.

Trust between the task force and the TST grew over time. At the beginning of the process, the TST members interviewed agreed that they had to run their decisions by the task force. As the TST demonstrated over time that it was fulfilling its responsibilities and making itself available for meetings, the task force trusted that the TST was acting in its best interest and gave the TST some autonomy to make minor adjustments without task force clearance.

Finally, informants that took part in the technical consultations perceived that the MOH was interested in learning from them because they were allowed to comment, and note takers were present for all consultation sessions. However, informants also perceived that trust was waning due to the length of time that had passed between the launch of the CIP process and action. Probing revealed that consultation participants expected the plan to be funded and initiated immediately upon the launch of the CIP because of donor presence at some of the consultations; however, they had not seen any CIP follow-up.

**Confidence.** Confidence was highest between the task force and the TST, which benefitted from clearly defined terms of reference, but was weak with regard to the consultations. Informants reported that, with few exceptions, minimal institutional agreements or arrangements were established for the CIP development process. For instance, informants from the task force and TST stated that roles and responsibilities between the task force and the TST were clearly defined and had a clear timeline, which helped govern the partnership. Furthermore, there was a discrete contract between one of the donors and the national consultant that allowed the latter to work on the CIP for a specific period of time. Overall, however, informants noted there were no contractual agreements between task force participants or between the task force and consultation participants. Also, no consultation participants received any partnership guidelines. DHOs interviewed suggested that their lack of engagement was a function of how Uganda works; they are expected to fall into line with MOH directives.

**Ownership.** Although ownership is not part of Brinkerhoff’s partnership evaluation framework, as mentioned earlier, it was a dominant theme that emerged from the key informant interviews. Within the task force, members perceived that the MOH took leadership of the CIP process and that other task force members were meant to support the MOH. The members thought this MOH leadership demonstrated that the CIP was important. Additionally, the task force and TST members interviewed recognized that even though the task force was committed to obtaining diverse perspectives, it had ultimate ownership over the decisions about what went into the CIP. The TST’s role was to collect and consolidate data from the consultations and present recommendations to the task force. These roles and responsibilities were clear from the start and gave each party the autonomy needed to fulfil its role. Nevertheless, informants noted that to sustain MOH ownership of the CIP’s execution, the MOH will need greater support from its senior management. For example, although the MOH had assigned 2 people from its Budget and Planning Office to work with the TST, their involvement was limited due to competing interests from their regular responsibilities.

In contrast, the DHOs interviewed did not perceive that they had ownership of the CIP. Although they were aware of several national and regional consultations, district representation was limited because the task force prioritized other groups—primarily NGOs seen to have technical expertise in key areas, such as youth, contraceptive security, and human resources. The DHO informants also noted that although the TST interviewed them while they were attending a national family planning conference, they were not included as experts in the technical areas of the CIP development. Rather, the DHOs interviewed believe that NGOs dominated the process, with little input from the districts, resulting in limited input from DHO implementers and allowing the NGOs to exercise too much influence over the direction of the plan.

District health officers lacked a sense of ownership over the CIP.

## DISCUSSION

Using a partnership evaluation framework highlighted several opportunities to strengthen the CIP development process in Uganda, which could have implications for CIP execution. Lessons learned from the process in Uganda could help inform practitioners in other countries developing family planning CIPs and other types of health strategies and policies. The CIP itself, by definition, clearly addresses funding and capacity building, and it includes a timeline for when activities should be executed.^15^ However, the development process in Uganda was focused on a short-term strategy that emphasized the CIP launch, rather than a long-term strategy that includes the development of key relationships that could serve as a springboard for the CIP’s execution—specifically, relationships between the task force and all stakeholders responsible for executing the CIP in the future.

### Advantages of Focusing on the CIP Launch

Because the CIP development process was a short-term strategy, the partnership was able to achieve several partnership prerequisites and success factors to a high degree, including a willingness to adapt to meet partnership needs, the existence of partnership champions, the ability to meet performance expectations, clear goals, senior management support, and partner compatibility. These factors were supported by a strong enabling environment that facilitated the establishment of the task force, a vibrant consultative process and culture of learning, active involvement of task force members, and availability of donor funding. Furthermore, the MOH’s ownership of the process demonstrated its commitment to developing the CIP and encouraged partners to participate fully in the entire process. However, clear roles and responsibilities were focused on developing the CIP—not necessarily on CIP execution.

Key informants from all 22 in-depth interviews perceived that power was not shared equally in the partnership, yet none of the task force members or consultation participants interviewed expected the MOH to share power because the end product was a government-owned policy. This sentiment was stated clearly by one informant who said, “The MOH took the lead. It’s a document of the MOH; we are supporting the MOH.” Thus, the willingness to share power might not be a necessary prerequisite when partners anticipate government ownership of the final product.

Willingness to share power might not be a necessary prerequisite for successful partnerships when partners anticipate government ownership of the final product.

### Challenges Due to Lack of Long-Term Strategy for Stakeholder Relationships

While there were advantages to keeping the task force focused on the goal of developing the CIP, this short-term focus resulted in challenges regarding the long-term strategy. For example, regarding the existence of champions, some people took extraordinary interest and action in supporting Uganda’s CIP in a variety of ways. TST members were technical champions; however, the TST is meant to only support CIP development, so its members should be seen mainly as short-term champions. It is not clear that these champions have sustained their efforts beyond the launch. For instance, the CIP calls for a National Steering and Coordination Committee for Family Planning-CIP,^15^ yet as of June 2015, there was no sign that it had been established, and the task force has not met since the CIP’s launch in November 2014 (personal communication with Dr. Nichole Zlatunich, Senior Program Advisor, Palladium, September 2015). This absence has heightened concerns about the CIP’s execution and whether key stakeholders will support its goals, strategies, and activities.

In fact, many key informants stated that momentum has already been lost, even as the national government and international partners recently completed an analysis of the financial gap for executing the CIP. For example, one informant stated, “Even with the CIP in place, we don’t know what the next step is. … The development process was vibrant. We are losing the vibrancy because the MOH is supposed to be driving the CIP, but I don’t know what is going on. I’ve asked, but I don’t have an answer.” Another informant noted, “If leadership is not taken up by the MOH, then there will be problems in implementation.” These concerns suggest that in relying on international support to develop the CIP, the national government was not fully prepared to provide the ongoing in-country leadership needed to execute it.

As to trust, by the time of the CIP launch, trust had been developed between the task force and the TST. One TST informant noted, “These relationships are very personal, and they’re built over time.” However, trust between the task force and the DHOs was not as strong. DHOs, who comprise a key group of implementers, felt that they had been excluded from the development process. One DHO informant commented, “It was not clear which role the districts were supposed to be playing.” This dynamic seems to be a reflection of the relationship between the national government and the districts in Uganda, where, despite a decentralized system of government, districts receive little autonomy or guidance.^21^ This situation could pose challenges to execution, as districts will have a key role to play in executing the CIP. If the task force had also chosen a long-term goal, e.g., improving the use of modern contraceptives, task force membership might have also included DHOs to ensure that implementers’ concerns were appropriately addressed in the CIP. The role of DHOs will be especially important when, as is always the case, changes occur in MOH staffing at the national level.

Because there was little deliberate focus on developing relationships that could support and strengthen CIP execution, partnership governing processes in Uganda remain informal or undocumented. Thus, confidence (in standard operating procedures and agreements between institutions) was mixed—no agreements came about for the partnership as a whole, despite the presence of some standard operating procedures, which applied mainly to the consultations but not to the relationships between partners. Additionally, there was no significant conflict during the development process, so it is difficult to assess what impact any future conflict may have on Uganda’s efforts to execute the CIP.

Similarly, the partners were able to meet expectations of CIP development, but informants were concerned about their ability to meet the expectations for execution as laid out in the CIP, such as securing funding. Uganda’s gap analysis found a total financial gap of about US$113 million across all 6 years of the CIP. Given that the total cost for the CIP is US$235.8 million, less than half of the costs in the CIP are covered by currently planned funding between 2015 and 2020.^16^ Addressing conflict and incompatibilities between partners and developing capacity along all levels of Uganda’s health system will need to be addressed. Although clear goals exist, they were focused on developing the CIP, not necessarily on how to prepare for execution. Anxieties that informants voiced about funding, decision making, and execution suggest that the factors above will likely become more critical once Uganda begins executing the CIP and relies on partners for discrete tasks laid out in the CIP. An informant stated, “The CIP has just been launched; the issue of reduced funding will be more relevant when implementation takes place.” These concerns will require sustained senior management support, including thought leadership, developing partnership guidelines and standard operating procedures, and mobilizing resources.

## RECOMMENDATIONS

**Use a long-term strategy in conjunction with a short-term strategy to set the stage for successful CIP execution.** The ultimate aim for the CIP is that it be executed effectively. Thus, its development process should be framed as the first phase of a long-term strategy or process, which could include a multiyear partnership to facilitate successful execution. Currently, the CIP development process is billed as a consultative process; in Uganda, this process was extensive. However, a consultative process may not be enough to ensure that the CIP is actually executed.

Uganda’s CIP calls for all stakeholders to work together to align their programs with the goals outlined in the CIP,^15^ implying an ongoing partnership between the Ugandan government and stakeholders. Moreover, there is precedent for including policy and strategy development as part of a partnership. For example, multi-organizational cross-sector social partnerships, which are becoming increasingly common as a way to address environmentally sustainable development, regularly create strategic plans as a first phase of a long-term partnership; once the plan is developed, the partnership continues with execution.^22^

**Use a partnership approach to help address key challenges.** The top portion of the Figure shows Uganda’s actual CIP development process along a basic timeline. The formal process included a 6-month development process, followed by a national launch and then the multiyear execution phase, which Uganda has just started. The bottom portion of the Figure shows an illustrative CIP development process that uses a partnership approach. With such an approach, the country lead and technical support partners would spend time before the development process officially begins to ask key questions that may not be asked when embarking on a short-term strategy focused on launching a policy document. Key questions when engaging in a long-term partnership might include the following: What is the ultimate goal? Given that goal, who should be the founding partners? What kinds of agreements between institutions are most appropriate for fostering partnership and demonstrating commitment? What kinds of structures and operating procedures should be in place to address execution challenges and disagreements between partners, and encourage dialogue?

**FIGURE:**
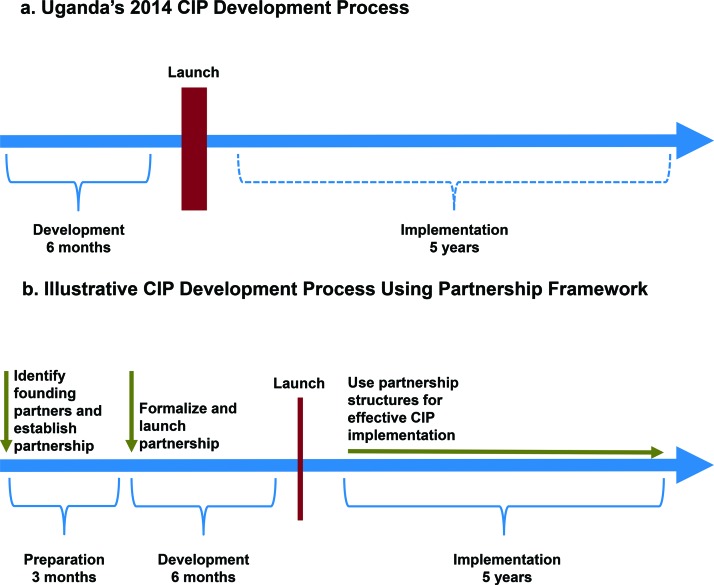
Uganda’s 2014 CIP Development Process Compared With an Illustrative CIP Development Process Using a Partnership Framework Abbreviation: CIP, costed implementation plan.

At the early stages, partnership guidance may be largely informal,^23^ yet the impact of emphasizing a partnership from the beginning can be significant simply because it demonstrates partner commitment to supporting the CIP in the long term and ensures a fully inclusive process. For example, in Uganda, including the CIP execution in the partnership’s terms of reference would help ensure that those first partners had included all implementers (DHOs, in Uganda’s case). Although it is too late to hold a consultation for the DHOs so they could contribute to the CIP, it is possible to select some DHO personnel to serve on the task force once the task force begins meeting again or to include selected DHO personnel on the Steering Committee, thus ensuring that the viewpoints of the district implementers are heard at the national level and facilitating communication between the national and district governments.^24^ While developing the CIP, the task force thus would also focus on formalizing the partnership relationships, establishing standard operating procedures, and identifying long-term roles and responsibilities so partner organizations, including districts, can begin making internal institutional arrangements to ensure they will be able to sustain their involvement.

Partnership thinking could also encourage country actors to begin contemplating early on how to build the data collection, costing, and analysis expertise capacities needed to monitor CIP performance and revise it as needed so that the country is not so reliant on international expertise. Once the CIP execution phase begins, partnership structures can be used to help with some of the key execution challenges identified by informants, mainly by providing a clear mechanism through which partners can raise issues and jointly address common challenges.

**Figure:**
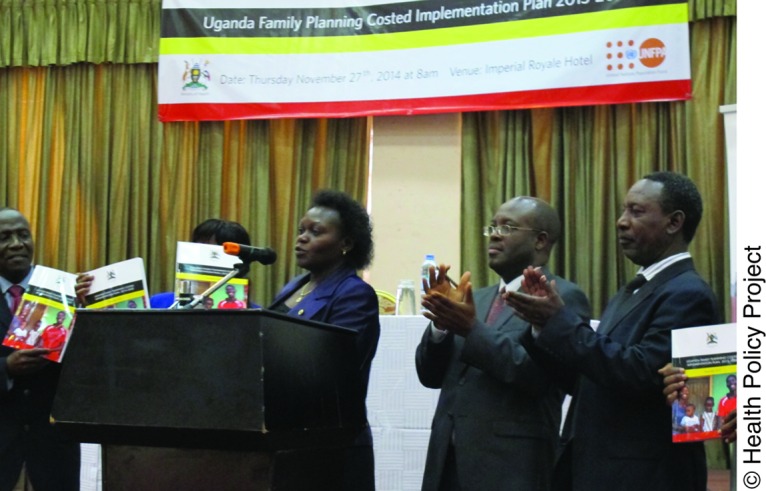
Ugandan officials celebrate the launch of the costed implementation plan (CIP).

**Create partnership structures that can hold partners accountable to their commitments.** Ideally, a partnership approach would result in structures that hold partners accountable to their commitments. A variety of practical steps can be taken to address accountability within partnerships. Having decided on partnership goals, developing a short list of indicators at the execution and organization levels to measure and report on partnership progress can help keep partners aligned and ensure accountability between them.^25^ Web-based technologies are providing more workable platforms to make this goal achievable. For example, DHIS2 is a web-based open-source health management information system being used around the world and within organizations for data management and analysis, monitoring and evaluation, and other tasks.^26^

Just as important is creating a “backbone support organization”—a separate organization and staff responsible for supporting CIP development and execution.^25^ More than a coordinating committee, it would be tasked with supporting partners in meeting their commitments, providing venues for dialogue, ensuring regular communication, and developing and maintaining a rigorous process for decision making.^25^ It would apply pressure to various partners when needed, mediate conflict when it arises, and “frame issues in a way that presents opportunities as well as difficulties.”^25^

## CONCLUSION

Although the CIP development process in Uganda was not executed as a partnership, using a partnership evaluation framework to assess its development shows that many partnership prerequisites and success factors existed and serves as an example for other countries that are developing long-term strategies that contribute to national and international family planning commitments and goals. Using a partnership framework may facilitate the next and most important step after development of the CIP—execution of the CIP. By ensuring that partners feel a long-term commitment, a partnership framework can result in institutions making arrangements for participation that extend beyond the CIP’s development and into its execution. Additionally, using the partnership framework can help establish transparent and accountable operating procedures and encourage building national capacity for data collection, analysis, and costing. Overall, including all relevant partners at the task force level can strengthen the partnership—specifically, relationships between DHOs and the MOH. However, a partnership approach, just like any other methodology or tool, is not a “silver bullet”—it would need to be executed with careful consideration and attention to the specific context to ensure it is used effectively.

While this article focuses on only one example of strategy development, many policy and strategy development efforts face some of the same funding, capacity, and sustainability challenges that became evident during the process of developing Uganda’s CIP.^27^ These challenges are long term and will always be present—the key is being able to address them when they arise during execution. Using a partnership approach can help ensure that supportive relationships exist. A strong partnership will leverage each member organization’s comparative advantage to fill gaps in funding and capacity as needed in a manner that strengthens the partnership and its member organizations and furthers policy and strategy execution.^1^^,^^23^

## Notes

**Peer Reviewed**
